# Evaluation of the Cost-effectiveness of Doublet Therapy in Metastatic *BRAF* Variant Colorectal Cancer

**DOI:** 10.1001/jamanetworkopen.2020.33441

**Published:** 2021-01-12

**Authors:** Kishan K. Patel, Stacey Stein, Jill Lacy, Mark O’Hara, Scott F. Huntington

**Affiliations:** 1 Department of Hematology/Oncology, Yale University School of Medicine, New Haven, Connecticut; 2 Department of Hematology/Oncology, University of Pennsylvania Perelman School of Medicine, Philadelphia; 3 Yale Cancer Outcomes, Public Policy and Effectiveness Research Center, New Haven, Connecticut

## Abstract

**Importance:**

The BEACON trial showed that combination therapy with encorafenib (BRAF inhibitor) and cetuximab (EGFR inhibitor) was associated with prolonged overall survival compared with standard chemotherapy in patients with metastatic *BRAF* variant colorectal cancer. However, the cost-effectiveness of using these agents in this clinical setting is unknown.

**Objective:**

To create a cost-effectiveness model to compare doublet therapy (encorafenib plus cetuximab) with standard chemotherapy (cetuximab plus irinotecan or cetuximab plus folinic acid, fluorouracil, and irinotecan) in treating patients with metastatic *BRAF* variant colorectal cancer.

**Design, Setting, and Participants:**

This economic evaluation constructed a Markov model to compare the lifetime cost and utility of doublet therapy and standard chemotherapy. Parametric survival modeling was used to extrapolate the effectiveness of each line of therapy from large clinical trials. One-way and probabilistic sensitivity analyses assessed the uncertainty in the model. Patients mirrored the cohorts in the BEACON trial: they had metastatic *BRAF* variant colorectal cancer and were followed up as they progressed through multiple lines of therapy, best supportive care, and death. Data collection and data analysis were performed from November 15, 2019, to July 14, 2020.

**Main Outcomes and Measures:**

The main outcome was the incremental cost-effectiveness ratio, which was calculated using the cumulative cost and effectiveness in quality-adjusted life years (QALYs), of doublet therapy compared with standard chemotherapy.

**Results:**

The model patient cohort had a mean age of 61 years, and 53% of the patients were women, 66% had 1 previous line of therapy, and 8% had high microsatellite instability. Doublet therapy was associated with an improvement of 0.15 QALYs compared with standard chemotherapy. However, the incremental cost of doublet therapy was $78 233, leading to an incremental cost-effectiveness ratio of $523 374 per QALY gained. Concomitant decreases in the price of encorafenib and cetuximab are needed to achieve cost-effectiveness at a willingness-to-pay threshold of $150 000 per QALY gained.

**Conclusions and Relevance:**

This study found that doublet therapy for metastatic *BRAF* variant colorectal cancer was unlikely to be cost-effective under current pricing. Cost-effectiveness needs to be considered in clinical trial design, particularly when combining new therapies with non-cost-effective treatments that are coadministered without a fixed duration.

## Key Points

**Question:**

Is the use of doublet therapy (encorafenib and cetuximab) compared with standard chemotherapy (with cetuximab and irinotecan-based regimens) a cost-effective treatment strategy in patients with metastatic *BRAF* variant colorectal cancer?

**Findings:**

In this economic evaluation of the cost-effectiveness of 2 treatment strategies, doublet therapy was associated with an incremental cost of $78 233, an incremental effectiveness of 0.15 quality-adjusted life years, and an incremental cost-effectiveness ratio of $523 374 per quality-adjusted life year gained.

**Meaning:**

Results of this study suggest that doublet therapy for metastatic *BRAF* variant colorectal cancer is unlikely to be a cost-effective treatment under current pricing.

## Teaser

This economic evaluation uses Markov modeling to identify and compare the cost and utility of doublet and standard treatments for patients with BRAF V600E variant colorectal cancer.

## Introduction

Colorectal cancer is the third most common cancer and is a leading cause of cancer death in the United States.^1^ *BRAF* (OMIM 164757) V600E–mutated colorectal cancer represents a discrete subtype that is found in approximately 10% of patients with metastatic disease.^2^ Previous work has demonstrated that patients with *BRAF* V600E sequence variation have inferior clinical outcomes compared with patients with *BRAF* wild-type,^3,4,5^ with a median overall survival of 4 to 6 months after failure of initial therapy.^2^

Attempts to treat *BRAF* variant colorectal cancer with single-agent BRAF inhibitors have achieved limited success.^6^ Evidence suggests that rapid feedback activation of the epidermal growth factor receptor (EGFR)^7^ may be a factor in the poor response to single-agent BRAF inhibitors. In clinical studies that have combined BRAF inhibitors with monoclonal antibodies against EGFR or mitogen-activated protein kinase kinase (MEK), BRAF inhibitors with monoclonal antibodies were shown to have improved response compared with BRAF inhibition alone.^8,9^ More recently, in a large phase 3 study (BEACON CRC [A Multicenter, Randomized, Open-Label, 3-Arm Phase 3 Study of Encorafenib + Cetuximab Plus or Minus Binimetinib vs Irinotecan/Cetuximab or Infusional 5-Fluorouracil (5-FU)/Folinic Acid (FA)/Irinotecan (FOLFIRI)/Cetuximab With a Safety Lead-in of Encorafenib + Binimetinib + Cetuximab in Patients With BRAF V600E-mutant Metastatic Colorectal Cancer]), patients with *BRAF* variant colorectal cancer were randomized to triplet therapy with encorafenib (BRAF inhibitor), binimetinib (MEK inhibitor), and cetuximab (EGFR inhibitor); doublet therapy with encorafenib and cetuximab; or standard chemotherapy with cetuximab plus irinotecan hydrochloride or cetuximab plus folinic acid, fluorouracil, and irinotecan (FOLFIRI).^2^ The BEACON study demonstrated improved outcomes with triplet and doublet therapy, with a median overall survival of 9.3 months for both regimens vs 5.9 months for standard chemotherapy.^10^ Based on these results, doublet therapy was approved by the US Food and Drug Administration for use in *BRAF* variant colorectal cancer.

Although doublet therapy has been associated with prolonged overall survival compared with standard chemotherapy,^2^ this treatment comes at a substantial cost. Encorafenib is priced at more than $96 000 per year. Furthermore, previous work has demonstrated that cetuximab is not cost-effective in the treatment of relapsed metastatic colorectal cancer.^11,12^ Therefore, we hypothesized that the addition of targeted therapy such as encorafenib to cetuximab would not result in a cost-effective treatment strategy because of the high cost of encorafenib and the baseline cost-ineffectiveness of cetuximab in the relapsed colorectal cancer setting.

## Methods

This economic evaluation study was deemed exempt from review per Yale University policy (45CFR46.101[b][4]) because it involves the collection or study of existing data. We followed the Consolidated Health Economic Evaluation Reporting Standards (CHEERS) reporting guideline.

We created a cost-effectiveness model to compare the use of doublet therapy (encorafenib plus cetuximab) with standard chemotherapy (cetuximab plus irinotecan or cetuximab plus FOLFIRI) in the treatment of metastatic *BRAF* V600E–mutated colorectal cancer beyond the first-line setting. Model patients mirrored the cohort that was assessed in the BEACON trial.^2^ Given that the BEACON study assessed the efficacy of doublet therapy in patients with known *BRAF* sequence variations, we assumed that all patients had already received next-generation sequencing (or other relevant molecular diagnostic tests) before being entered into our model.

### Model Construction

**Figure 1.**
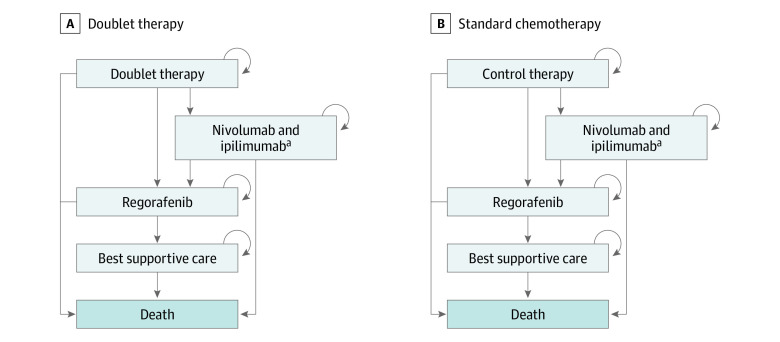
Diagram of Markov Models Markov models for individuals who received doublet therapy (A) or standard chemotherapy (B). ^a^Only patients with high-microsatellite instability (8% of the simulated cohort in the base case analysis) received therapy with nivolumab and ipilimumab.

This cost-effectiveness analysis was conducted using a Markov model (Figure 1). Patients who were entered into the model had metastatic *BRAF* V600E–mutated colorectal cancer that was progressing after 1 or 2 previous lines of therapy and could receive either doublet therapy or standard chemotherapy until progression. After progression, patients with high microsatellite instability received checkpoint inhibitor therapy with nivolumab plus ipilimumab. Patients without high microsatellite instability or patients whose cancer progressed on checkpoint inhibitor therapy subsequently received regorafenib. Dose and administration of each line of treatment were based on the respective clinical trial of these treatments.^2,13,14^ Individuals whose cancer progressed on regorafenib ultimately entered a best-supportive-care health state before death.

We constructed the Markov model with a 1-month Markov cycle and a lifetime horizon. The model was used to estimate the cumulative cost and effectiveness, in quality-adjusted life years (QALYs), of each treatment strategy. We used these outputs to calculate an incremental cost-effectiveness ratio (ICER) for doublet therapy, which reflects the cost in 2019 US dollars for each additional QALY gained as a result of treatment. This analysis was conducted from a US health care perspective and with a willingness-to-pay threshold of $150 000 per QALY gained and an annual discount rate of 3% for both costs and utilities.^15,16^ The Markov model was created using TreeAge Pro, version 2021 (TreeAge Software), and parametric survival modeling was performed using R, version 1.2.5033 (R Foundation for Statistical Computing), and Stata, version 16.1 (StataCorp LLC).

### Transition Probabilities

Base case estimates for transition probabilities are provided in Table 1. Using published extrapolation techniques, we ascertained the progression rates for standard chemotherapy, regorafenib, and nivolumab plus ipilimumab from the progression-free survival curves of each respective clinical trial.^19,20^ Recreated individual patient-level data for standard chemotherapy and regorafenib monotherapy were best fit using a Weibull distribution, whereas data for nivolumab plus ipilimumab were best fit using a Gompertz distribution (eFigures 1 to 3 in the Supplement). Transition probabilities for doublet therapy were identified by using the reported hazard ratio (HR) with reference to standard chemotherapy. The percentage of model patients who were to receive checkpoint inhibitor therapy before regorafenib was based on the mean percentage of patients with high microsatellite instability in the BEACON trial.^2^

**Table 1.** Model Clinical Parameters

Parameter	Estimate (range)	Study or data source
PFS for standard chemotherapy	Weibull: λ = 0.2415957; κ = 1.280836	Kopetz et al,^2^ 2019
HR for doublet therapy	0.40 (0.31-0.52)	Kopetz et al,^2^ 2019
PFS for regorafenib	Weibull: λ = 0.1579101; κ = 1.439624	Grothey et al,^13^ 2013
PFS for nivolumab plus ipilimumab	Gompertz: λ = 0.0674932; γ = –0.1807722	Overman et al,^14^ 2018
Probability of background death, %	NA	Arias et al,^17^ 2019
Time spent in best supportive care state, mo	0.5	Model calibration
Discount rate, annual, %	3.0 (1.5-6.0)	Sanders et al,^16^ 2016; Huntington et al,^18^ 2018
Probability of receiving FOLFIRI in standard chemotherapy (vs irinotecan hydrochloride), %	50 (0-100)	Expert opinion
Probability of discontinuing treatment because of AE, %		
Standard chemotherapy	5.2 (2.6-7.8)	Kopetz et al,^2^ 2019
Doublet therapy	5.1 (2.5-7.6)	Kopetz et al,^2^ 2019
Regorafenib	8.4 (4.2-12.6)	Grothey et al,^13^ 2013
Nivolumab plus ipilimumab	13.4 (6.7-20.2)	Overman et al,^14^ 2018
Probability of treatment mortality because of AE, %		
Standard chemotherapy	0.5 (0.3-0.8)	Kopetz et al,^2^ 2019
Doublet therapy	0.5 (0.2-0.7)	Kopetz et al,^2^ 2019
Regorafenib	1.6 (0.8-2.4)	Grothey et al,^13^ 2013
Patients with MSI-H in each treatment strategy, %	8 (4-12)	Kopetz et al,^2^ 2019
Median number of nivolumab doses received for patients with MSI-H	24 (18-30)	Overman et al,^14^ 2018
Median starting age of population, y	61 (26-91)	Kopetz et al,^2^ 2019
Male patients in cohort, %	47	Kopetz et al,^2^ 2019
Patients with 1 previous line of therapy, %	66	Kopetz et al,^2^ 2019

Abbreviations: AE, adverse event; FOLFIRI, folinic acid, fluorouracil, and irinotecan; HR, hazard ratio; MSI-H, high microsatellite instability; NA, not applicable; PFS, progression-free survival.

We incorporated in the model the discontinuation of each line of treatment owing to adverse events or treatment mortality, with probabilities derived from the literature.^2,13,14^ We assumed that adverse event–related treatment discontinuation occurred within the first 2 months of treatment, to account for the greater frequency of severe toxic effects in that time frame.^13^ A background mortality rate was identified from US life tables.^17^ The amount of time spent in the best-supportive-care state was established using model calibration. During calibration, the monthly transition probability of death from best supportive care was varied incrementally. The value that allowed the model median overall survival of each treatment strategy to most closely reflect the updated results of the BEACON trial was chosen for inclusion in the base case model.^10^

### Costs and Utility Values

Costs that were included in the model are outlined in Table 2. The costs of intravenous medication, including cetuximab, irinotecan, fluorouracil, folinic acid, nivolumab, and ipilimumab, were derived from the October 2019 Centers for Medicare & Medicaid Services (CMS) Average Sales Price Drug Pricing Files.^24^ The cost of chemotherapy administration was based on the 2019 CMS Physician Fee Schedule.^25^ We assumed a mean total body surface area of 1.7 m^2^ and a mean weight of 70 kg, and then we rounded up to the nearest single-use vial size available for each dose administered.^26^ Because the BEACON trial did not report the percentage of patients in the standard chemotherapy group who received cetuximab plus irinotecan vs cetuximab plus FOLFIRI, we assumed that 50% of patients received each regimen in the base case analysis. The costs of oral targeted therapy agents, including encorafenib and regorafenib, were obtained from the CMS Plan Finder tool.^27,28^ To accommodate a health care perspective, we included both third-party payer and patient out-of-pocket costs in the calculations.

**Table 2.** Model Costs and Utility Values

Treatment	Baseline cost (range), $	Study or data source
Encorafenib, 300 mg once daily, per mo	8049.80	CMS Plan Finder
Regorafenib, 160 mg once daily, per mo	19 536.48	CMS Plan Finder
Cetuximab, 500 mg, per dose	3102.40	HCPCS J9055
Irinotecan, 320 mg, per dose	39.58	HCPCS J9206
Fluorouracil, 5000 mg, per dose	15.50	HCPCS J9190
Folinic acid, 350 mg, per dose	21.13	HCPCS J0640
Nivolumab, 240 mg, per dose	6673.20	HCPCS J9299
Ipilimumab, 100 mg, per dose	15 312.80	HCPCS J9228
Routine office visit	112.80 (105.32-152.91)	*CPT* 99215
Chemotherapy IV infusion, first hour	143.08 (124.35-188.20)	*CPT* 96413
Chemotherapy IV infusion, additional hour	30.99 (27.49-39.41)	*CPT* 96415
Chemotherapy IV infusion, additional sequence	69.20 (60.46-90.25)	*CPT* 96417
Preinfusion medication	12.30	Barnes et al,^21^ 2018
CBC with differential	8.63	*CPT* 85025
Carcinoembryonic antigen	21.07	*CPT* 82378
Alanine aminotransferase	5.75	*CPT* 84460
Aspartate aminotransferase	5.75	*CPT* 84450
Lipase	7.65	*CPT* 83690
CT abdomen/pelvis with contrast	323.99 (288.42-417.08)	*CPT* 74177
End-of-life care	42 740.36 (21 370.18-64 110.54)	Chastek et al,^22^ 2012
Metastatic colorectal cancer, utility (range)	0.66 (0.59-0.735)	Goldstein et al,^23^ 2015

Abbreviations: CBC, complete blood count; CMS, Centers for Medicare & Medicaid Services; *CPT*, *Current Procedural Terminology*; CT, computed tomography; HCPCS, Healthcare Common Procedure Coding System; IV, intravenous.

We estimated that patients would receive routine follow-up every month, which included a physician office visit and standard laboratory tests such as carcinoembryonic antigen, complete blood count, liver function, and serum lipase tests. These costs were obtained from the 2019 CMS Physician Fee Schedule and 2019 CMS Q4 Medicare Clinical Laboratory Fee Schedule.^25,29^ We assumed that patients would receive a computed tomography scan of the abdomen and pelvis every other month. The costs of grade 3 or 4 adverse events were also incorporated into the model; each adverse event was managed according to published guidelines and past cost-effectiveness analyses^23,30,31^ (eTable in the Supplement). The cost of end-of-life care was derived from the literature.^22^ All costs were converted to 2019 US dollars using the Personal Consumption Expenditures–Health index.^32^

The base case model used a utility value of 0.66 for each health state apart from death. This utility value was based on a previous cost-effectiveness analysis^23^ that used the mean EQ-5D index score of patients in the CORRECT (A Randomized, Double-Blind, Placebo-Controlled Phase 3 Study of Regorafenib Plus BSC Versus Placebo Plus BSC in Patients With Metastatic Colorectal Cancer [CRC] Who Have Progressed After Standard Therapy) trial,^13^ which examined the efficacy of the multikinase inhibitor regorafenib in metastatic colorectal cancer.

### Statistical Analysis

Data collection and data analysis were performed from November 15, 2019, to July 14, 2020. We conducted sensitivity analyses to evaluate the uncertainty in the model. During 1-way sensitivity analyses, individual parameters were varied across the ranges detailed in Tables 1 and 2 to ascertain their role in the ICER. Utility values and HRs were varied across their 95% CIs. Other transition probabilities were varied within a 50% range. During probabilistic sensitivity analyses, each parameter was represented using a distribution and 10 000 Monte Carlo simulations were performed using random sampling from the distribution of each model input each time. Costs were described by γ distributions, and probabilities and utility values were represented by β distributions.^33^

In addition, we conducted threshold analyses in which the prices of encorafenib and cetuximab were decreased, either individually or simultaneously, to identify the changes in the ICER of doublet therapy. We also performed a scenario analysis in which patients whose cancer progressed on doublet therapy or standard chemotherapy subsequently received best supportive care rather than undergoing additional treatment with regorafenib and/or nivolumab plus ipilimumab.

## Results

The model patient cohort had a mean age of 61 years, and 53% of the patients were women and 47% were men, 66% had 1 previous line of therapy, and 8% had high microsatellite instability. Use of doublet therapy was associated with an improvement of 0.15 QALYs compared with use of standard chemotherapy (1.07 vs 0.92 QALYs) (Table 3). However, doublet therapy was associated with substantially greater lifetime health care costs than standard chemotherapy ($238 276 vs $160 043), with an incremental cost of $78 233. Therefore, the ICER for doublet therapy was $523 374 per QALY gained compared with the strategy of using standard chemotherapy with cetuximab plus irinotecan or cetuximab plus FOLFIRI.

**Table 3.** Baseline Cost-effectiveness Analysis

Base case model						PSA model
Strategy	Cost, US $	Incremental cost, US $	Effectiveness, QALY	Incremental effectiveness, QALY	ICER, $/QALY	ICER 95% CI, $/QALY
Doublet therapy	238 276	78 233	1.07	0.15	523 374	450 581-638 854
Standard chemotherapy	160 043	NA	0.92	NA	NA	NA

Abbreviations: ICER, incremental cost-effectiveness ratio; NA, not applicable; PSA, probabilistic sensitivity analysis; QALY, quality-adjusted life year.

**Figure 2.**
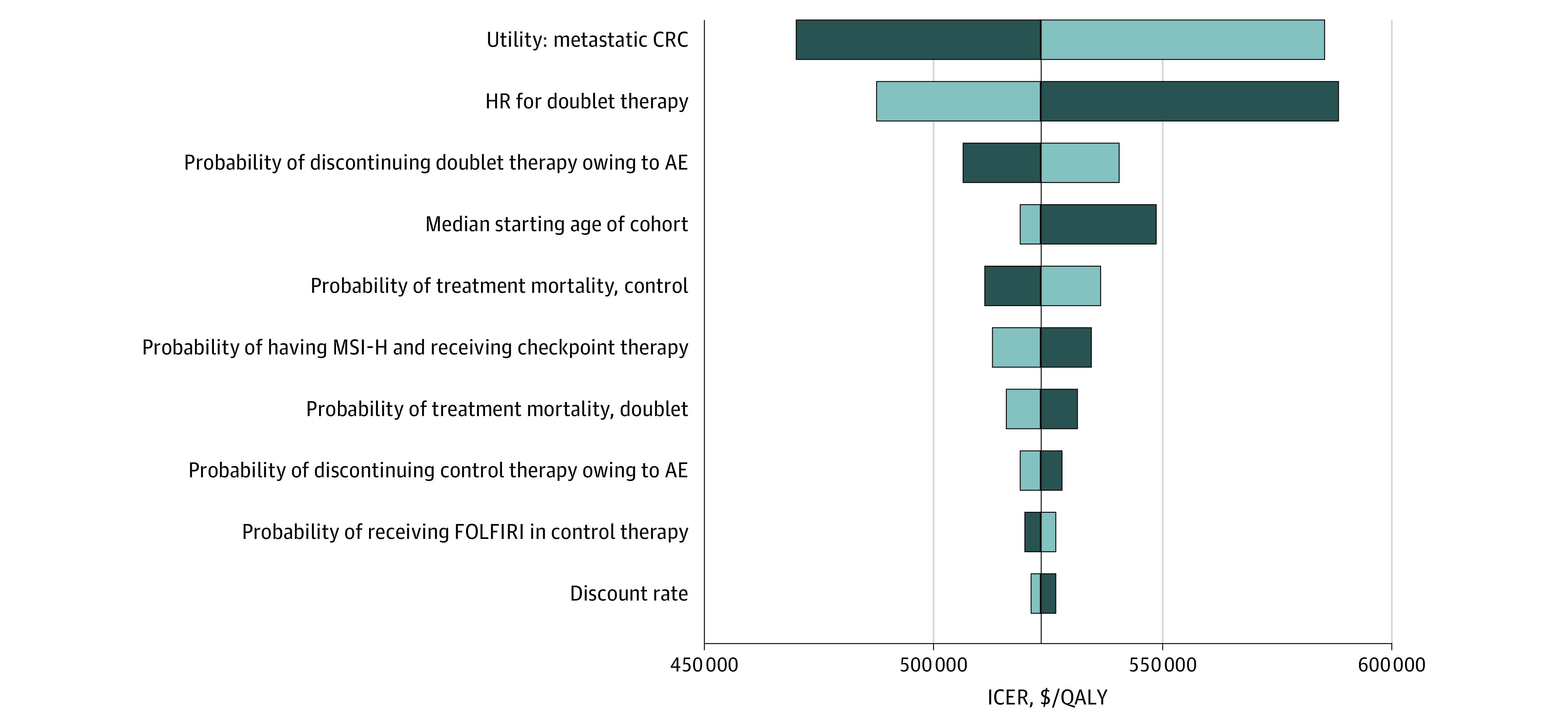
One-Way Sensitivity Analyses All model inputs with ranges outlined in Tables 1 and 2 were evaluated during 1-way sensitivity analyses; however, only parameters that produced a change greater than US $5000 per quality-adjusted life year (QALY) when evaluated across their entire range were included in the Tornado diagram. AE indicates adverse event; CRC, colorectal cancer; FOLFIRI, folinic acid, fluorouracil, and irinotecan; HR, hazard ratio; ICER, incremental cost-effectiveness ratio; and MSI-H, high microsatellite instability. Light blue bars represent the lower value in the range, whereas dark blue bars represent the higher value.

The ICER for doublet therapy was most sensitive to the utility value of metastatic colorectal cancer (Figure 2). For instance, decreasing the utility value from 0.66 to 0.59 increased the ICER of doublet therapy to $585 465 per QALY gained, whereas increasing the utility value to 0.735 decreased the ICER to $469 968 per QALY gained. Other model inputs that substantially changed the ICER included the progression-free survival HR of doublet therapy compared with standard chemotherapy, the probability of discontinuing treatment owing to adverse events, and the median starting age of the cohort. All ICERs during 1-way sensitivity analyses remained above the willingness-to-pay threshold of $150 000 per QALY gained. During probabilistic sensitivity analyses, 100% of iterations produced ICERs that were greater than the willingness-to-pay threshold (eFigure 4 in the Supplement).

Threshold analysis showed that doublet therapy could not achieve cost-effectiveness regardless of price decreases in oral targeted therapy. Reducing the price of encorafenib to $0 per month lowered the ICER for doublet therapy to $233 304 per QALY gained. Simultaneous decreases in the price of encorafenib and cetuximab were needed to achieve cost-effectiveness at a willingness-to-pay threshold of $150 000 per QALY gained. For example, if cetuximab was discounted by 50% (ie, $32.02 per 10 mg vs current $64.05 per 10 mg), a 90% discount for encorafenib would produce an ICER of $150 000 per QALY gained for doublet therapy (eFigure 5 in the Supplement).

We also included a scenario analysis in which patients whose cancer progressed on doublet therapy or standard chemotherapy proceeded to best supportive care without receiving further treatment. This scenario minimally changed the model, with an ICER of $501 447 per QALY gained for doublet therapy vs standard chemotherapy.

## Discussion

The BEACON trial showed that combination targeted therapy with encorafenib and cetuximab was associated with prolonged survival in patients with metastatic *BRAF* variant colorectal cancer.^2^ However, the results of this economic evaluation suggested that doublet therapy was unlikely to represent a cost-effective treatment strategy compared with cetuximab and chemotherapy, with an ICER exceeding $500 000 per QALY gained. Furthermore, results of this study demonstrated that a price decrease for encorafenib alone was insufficient to achieve cost-effectiveness because of the baseline low economic value of cetuximab in this clinical setting.^11,12^ Because doublet therapy allows patients to remain on the cost-ineffective cetuximab for longer periods, this strategy was unable to reach cost-effectiveness in the present model even if encorafenib was supplied to patients for free.

The real-world performance of standard chemotherapy in metastatic *BRAF* variant colorectal cancer may be superior to that reported in the BEACON trial. As critics of the BEACON study have noted,^34^ the control group of the BEACON trial may not reflect the optimal treatment strategy for some patients because it included only irinotecan or FOLFIRI rather than allowing for oxaliplatin-based therapy, despite a substantial proportion of patients who received previous irinotecan-based treatment. In the present model, we conservatively estimated survival of patients in the standard chemotherapy group using the data provided by the BEACON trial; therefore, the incremental effectiveness of doublet therapy may be inflated compared with standard clinical practice in which exposure to previous therapy could more rationally inform subsequent chemotherapy selection. Thus, this model likely provides conservative estimates of the cost-effectiveness of doublet therapy compared with available standard chemotherapy.

To our knowledge, this study is the first to examine the cost-effectiveness of doublet therapy in the treatment of metastatic *BRAF* variant colorectal cancer in the United States. The results suggest that doublet therapy is unlikely to achieve cost-effectiveness without reducing the price of cetuximab, findings that are consistent with those of previous studies that found high ICERs when incorporating cetuximab into the treatment of metastatic colorectal cancer.^11,12^ Even a scenario in which encorafenib was administered without cost produced an ICER for doublet therapy that exceeded $200 000 per QALY gained. Without broad changes to drug pricing, the costs associated with doublet therapy are unlikely to represent reasonable value for the setting of relapsed metastatic *BRAF* variant colorectal cancer.

### Strengths and Limitations

This study has several strengths. First, the cost-effectiveness model was based on a large randomized clinical trial that directly compared doublet therapy with standard chemotherapy.^2^ As a result, we were able to model transition probabilities for each of the treatment strategies, and we could account for uncertainty by varying the HR of doublet therapy across the 95% CI provided by the BEACON trial. Second, we incorporated contemporary data in the treatment of metastatic colorectal cancer, such as the use of checkpoint inhibitor therapy for patients with high microsatellite instability^14^ and the use of regorafenib in individuals for whom all other lines of treatment had failed.^13^ Third, we accounted for drug waste by rounding up to the closest single-use vial size for each administered dose of chemotherapy, a practice that reflects the most accurate costs of drug administration and has the potential to substantially change reported ICERs.^26^ Fourth, we incorporated into the model the impact of serious adverse events and accounted for the cost of medical management.

This study also has several limitations. First, although the transition probabilities were derived from large clinical trials using parametric survival modeling, the post-trial outcomes for patients were uncertain. Second, limited data were available on the survival of patients with *BRAF* variant colorectal cancer who received regorafenib. The model used data from the CORRECT trial, which contained only a small percentage of patients with *BRAF* sequence variations^13^; as a result, the model overall survival may overestimate the overall survival seen in this patient population. However, given that regorafenib outcomes were identical across each treatment group, this limitation is not expected to substantially affect the incremental effectiveness or the overall ICER for doublet therapy.

## Conclusions

Although the BEACON trial showed an improvement in overall survival associated with doublet therapy for metastatic *BRAF* variant colorectal cancer, the model created in this economic evaluation suggested that doublet therapy was unlikely to be cost-effective. Doublet combination therapies that lead to longer administration of cetuximab were unable to be cost-effective in this model even when encorafenib was provided without cost. In an era of surging health care costs and limited health care resources, cost-effectiveness needs to be considered in clinical trial design, particularly when combining new therapies with non-cost-effective treatments that are coadministered without a fixed duration.

## Notes

Supplement. **eTable.** Costs Due to Adverse Events **eFigure 1.** Parametric PFS Curves for Standard Chemotherapy and Doublet Therapy **eFigure 2.** Parametric PFS Curve for Regorafenib **eFigure 3.** Parametric PFS Curve for Nivolumab Plus Ipilimumab **eFigure 4.** Probabilistic Sensitivity Analysis **eFigure 5.** Two-Way Sensitivity Analysis With Costs of Cetuximab and Encorafenib **eReferences**
