# Supplementary material for: Evaluation of the Cost-effectiveness of Doublet Therapy in Metastatic BRAF Variant Colorectal Cancer
Source: JAMA Netw Open. 2021 Jan 12;4(1):e2033441. doi: 10.1001/jamanetworkopen.2020.33441 (PMC7804917; doi:10.1001/jamanetworkopen.2020.33441)
Supplement: Supplement. — eTable. Costs Due to Adverse Events eFigure 1. Parametric PFS Curves for Standard Chemotherapy and Doublet Therapy eFigure 2. Parametric PFS Curve for Regorafenib eFigure 3. Parametric PFS Curve for Nivolumab Plus Ipilimumab eFigure 4. Probabilistic Sensitivity Analysis eFigure 5. Two-Way Sensitivity Analysis With Costs of Cetuximab and Encorafenib eReferences [file jamanetwopen-e2033441-s001.pdf]

Supplementary Online Content

Patel KK, Stein S, Lacy J, O'Hara M, Huntington SF. Evaluation of the cost-effectiveness of doublet therapy in metastatic *BRAF* variant colorectal cancer. *JAMA Netw Open*. 2021;4(1):e2033441. doi:10.1001/jamanetworkopen.2020.33441

eTable. Costs Due to Adverse Events

eFigure 1. Parametric PFS Curves for Standard Chemotherapy and Doublet Therapy

eFigure 2. Parametric PFS Curve for Regorafenib

eFigure 3. Parametric PFS Curve for Nivolumab Plus Ipilimumab

eFigure 4. Probabilistic Sensitivity Analysis

eFigure 5. Two-Way Sensitivity Analysis With Costs of Cetuximab and Encorafenib

eReferences

This supplementary material has been provided by the authors to give readers additional information about their work.

©2021 Patel KK et al. *JAMA Network Open*

eTable. Costs Due to Adverse Events

Treatment	Adverse Event (Grade 3 or 4)	Percentage of Patients Experiencing AE	Monthly Probability	Cost of AE (per event)	**Total Cost (monthly)**
**Regorafenib**	Fatigue	10%	0.060095283	$0.00	$48.73
Median Duration of Therapy: 1.7 months[1]	Hand-foot skin reaction	17%	0.103812509	$296.30	
	Diarrhea	8%	0.047864579	$141.80	
	Rash	6%	0.035742878	$173.30	
	Hypertension	7%	0.041790311	$119.30	
**Doublet Therapy**	Fatigue	4%	0.009281264	$0.00	$1.45
Median Duration of Therapy: 4.38 months[2]	Diarrhea	2%	0.004604092	$141.80	
	Nausea	1%	0.002293075	$119.30	
	Abdominal Pain	2%	0.004604092	$112.80	
**Standard Chemotherapy**	Fatigue	4%	0.024992033	$0.00	$13.24
Median Duration of Therapy: 1.61 months[2]	Diarrhea	10%	0.063235647	$141.80	
	Nausea	1%	0.006211835	$119.30	
	Abdominal Pain	5%	0.031301482	$112.80	
**Nivolumab + Ipilimumab**	Fatigue	2%	0.001753691	$0.00	$2.99
Median Duration of Therapy: 11.51 months[3]	Diarrhea	2%	0.001753691	$141.47	
	Hepatitis	11%	0.01007349	$187.47	
	Pancreatitis	4%	0.003540373	$172.07	
	Rash	2%	0.001753691	$141.47	

©2021 Patel KK et al. *JAMA Network Open*

eFigure 1. Parametric PFS Curves for Standard Chemotherapy and Doublet Therapy

A Weibull distribution was used for standard chemotherapy, and the PFS curve for doublet therapy was based on the reported hazard ratio. KM = Kaplan-Meier; HR = hazard ratio.

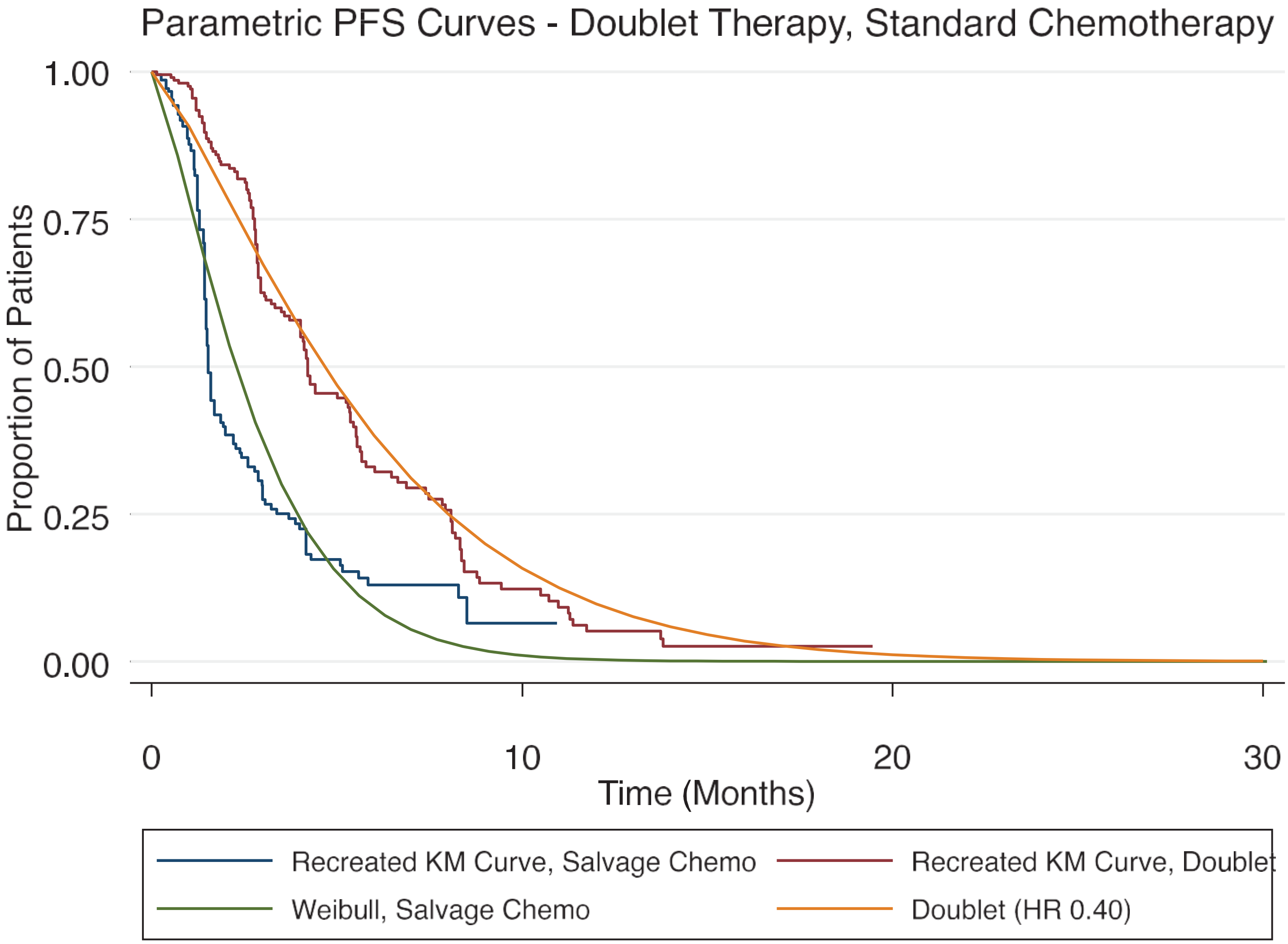

©2021 Patel KK et al. *JAMA Network Open*

eFigure 2. Parametric PFS Curve for Regorafenib

KM = Kaplan-Meier.

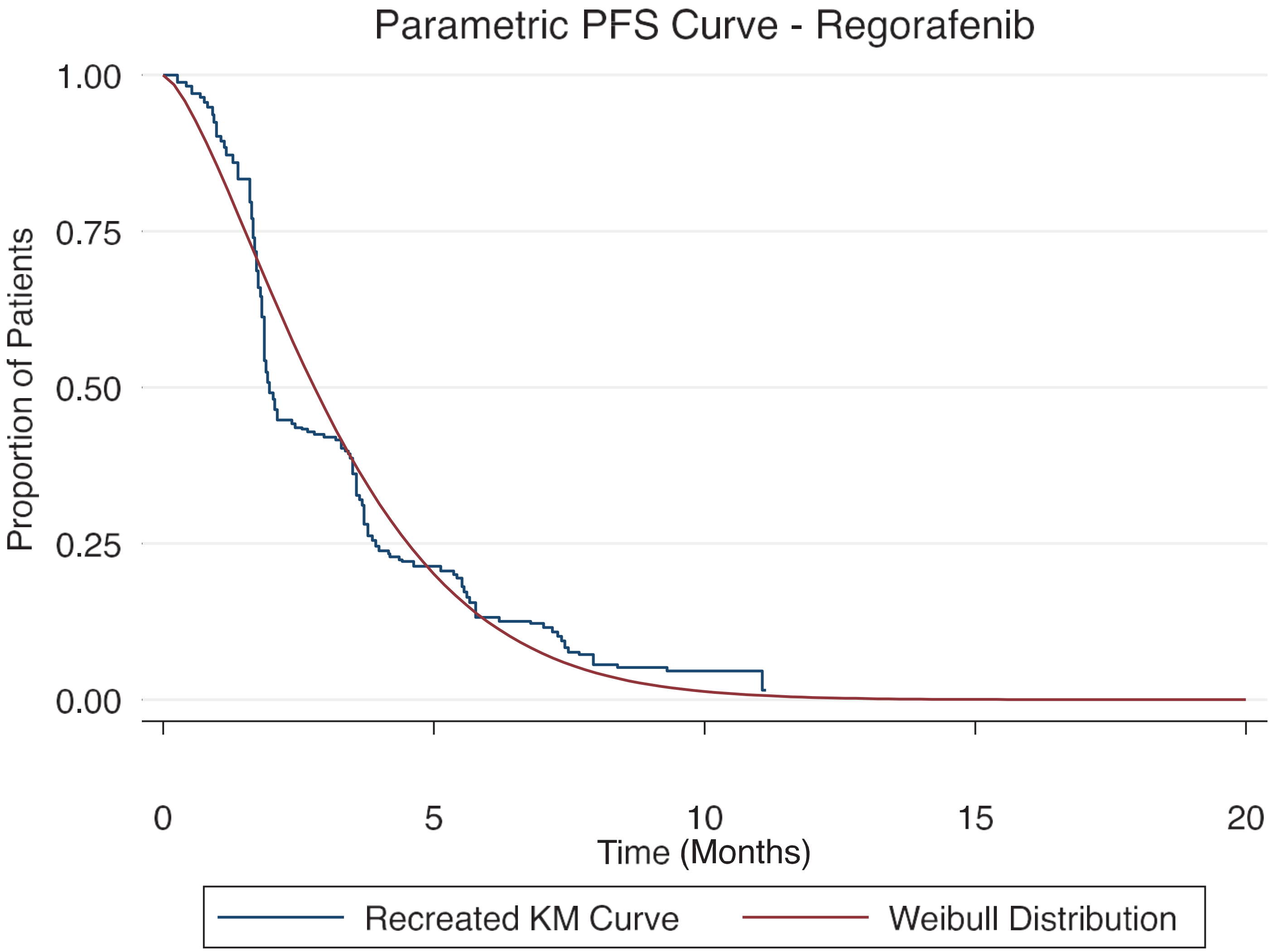

©2021 Patel KK et al. *JAMA Network Open*

eFigure 3. Parametric PFS Curve for Nivolumab Plus Ipilimumab

KM = Kaplan-Meier.

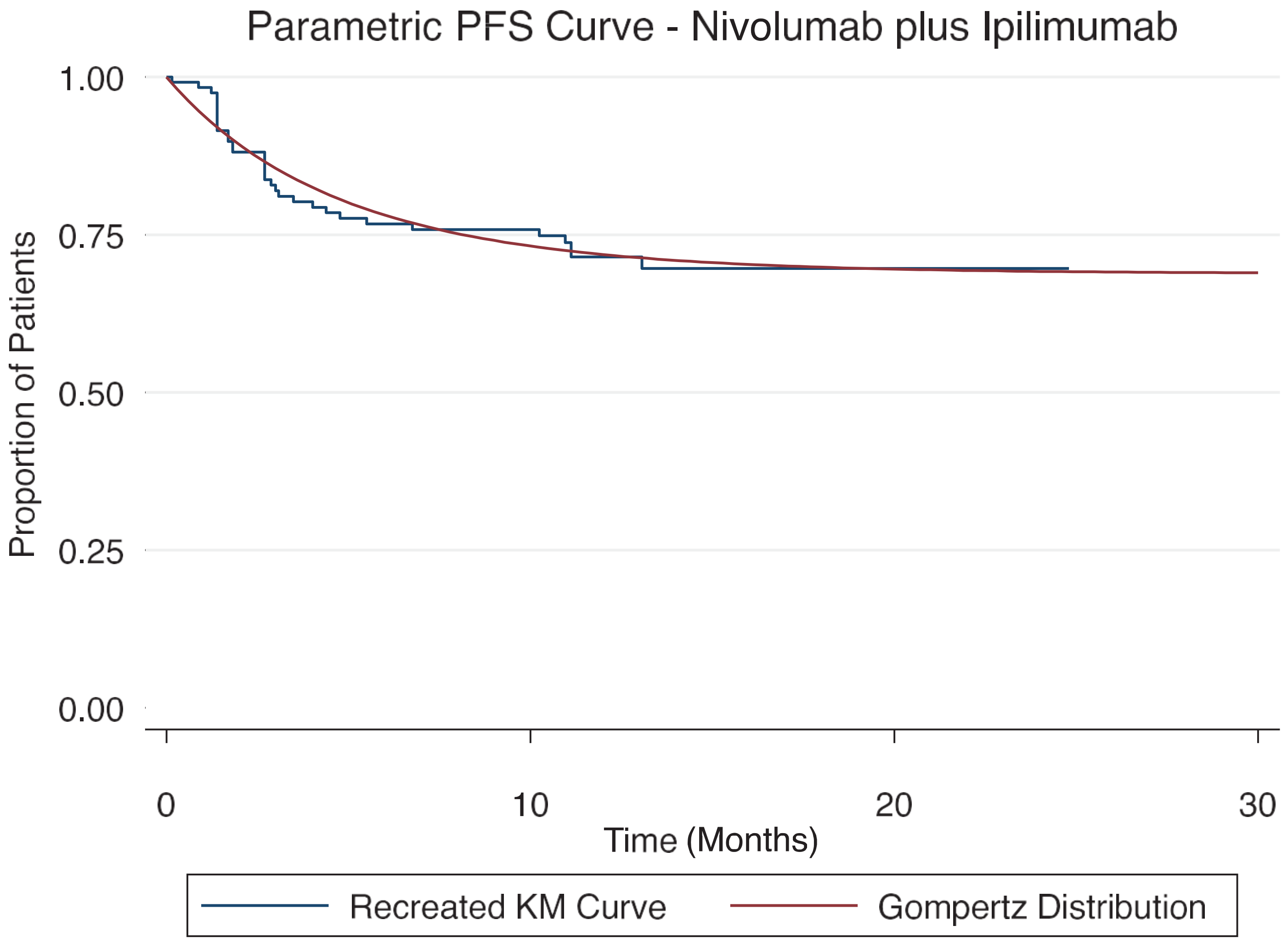

©2021 Patel KK et al. *JAMA Network Open*

eFigure 4. Probabilistic Sensitivity Analysis

Results of the probabilistic sensitivity analyses are based on 10,000 Monte Carlo simulations.

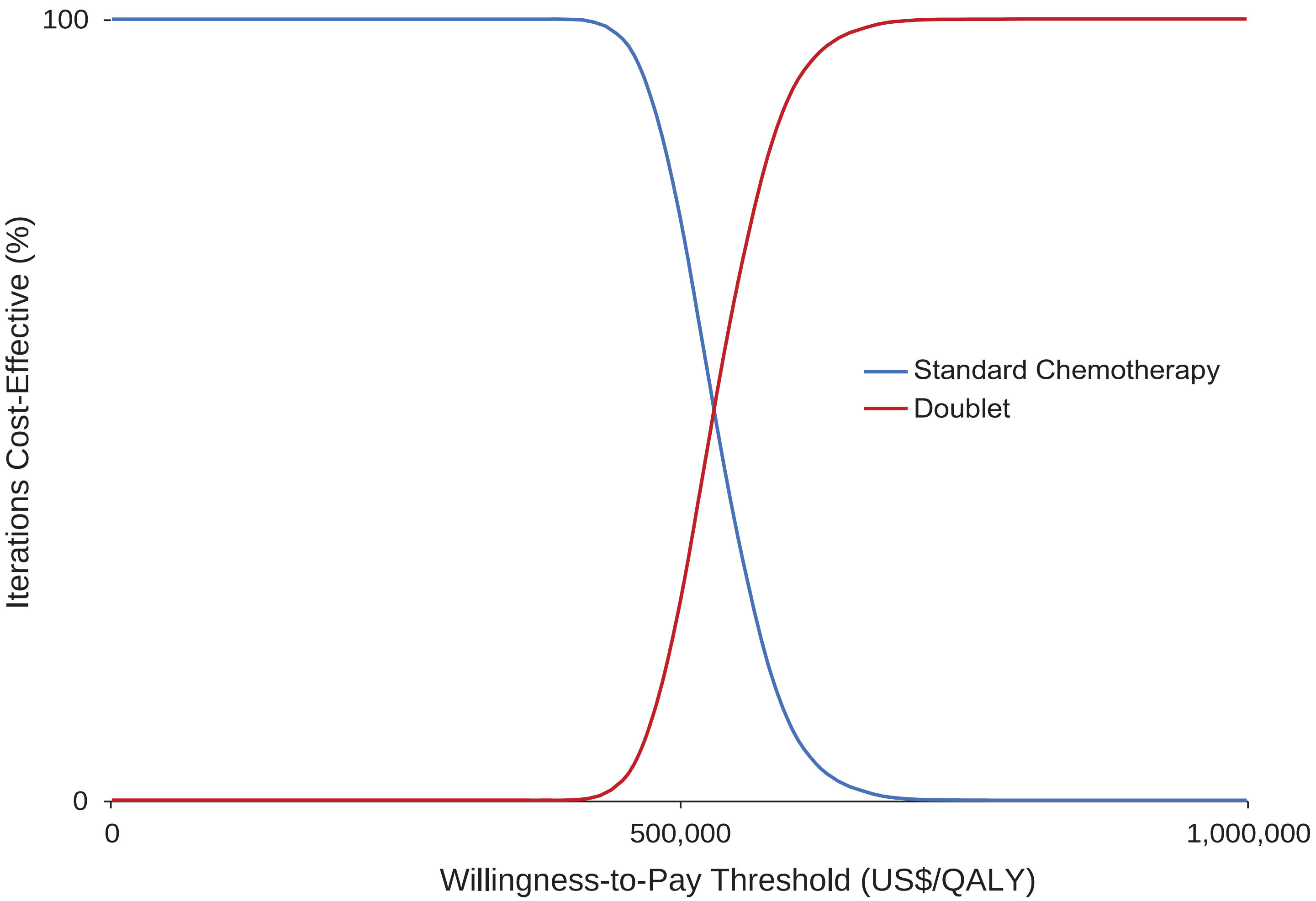

©2021 Patel KK et al. *JAMA Network Open*

eFigure 5. Two-Way Sensitivity Analysis With Costs of Cetuximab and Encorafenib

Red area indicates cost-effectiveness at a willingness-to-pay threshold of $150,000/QALY; blue area represents cost-ineffectiveness.

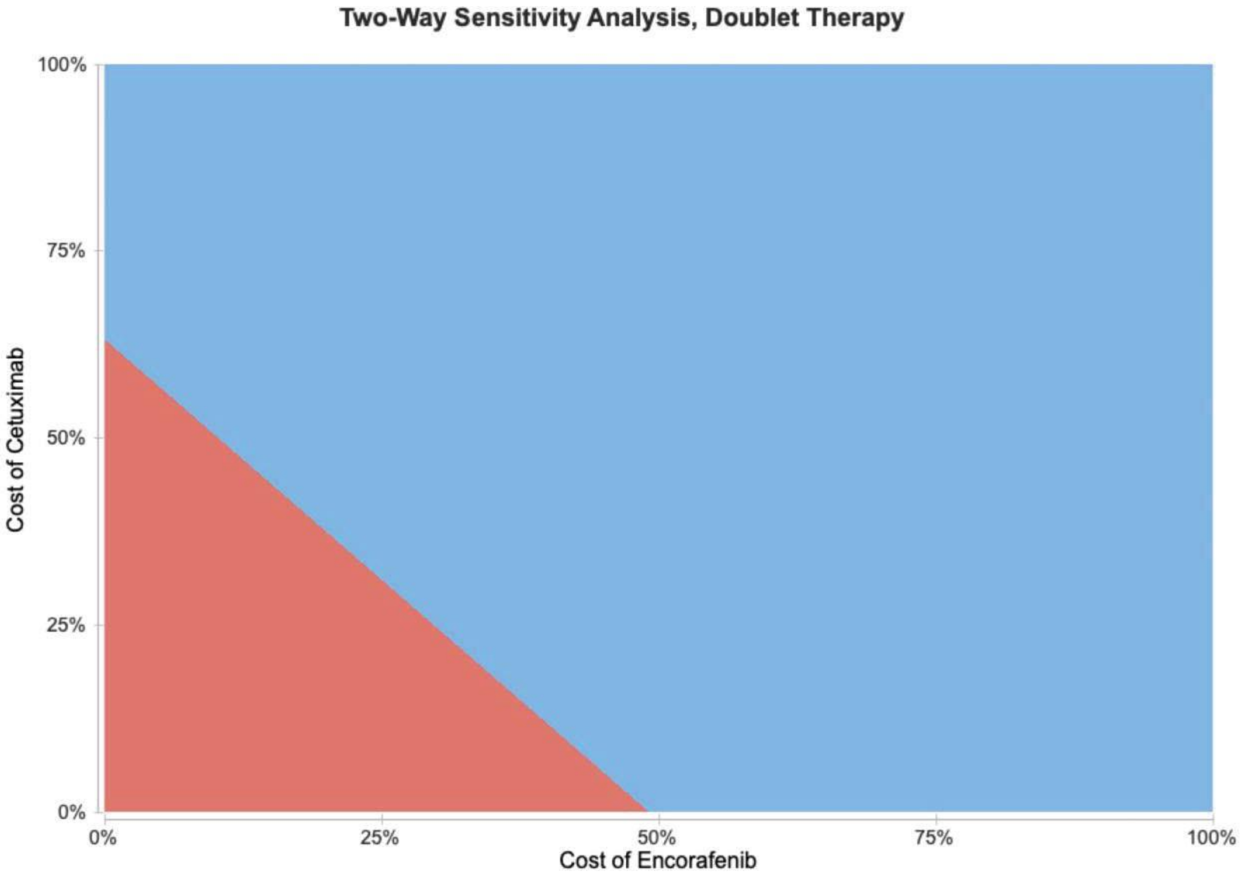

©2021 Patel KK et al. *JAMA Network Open*

eReferences

1. Grothey A, Van Cutsem E, Sobrero A, et al. Regorafenib monotherapy for previously treated metastatic colorectal cancer (CORRECT): an international, multicentre, randomised, placebo-controlled, phase 3 trial. *Lancet.* 2013;381(9863):303-312.
2. Kopetz S, Grothey A, Yaeger R, et al. Encorafenib, Binimetinib, and Cetuximab in BRAF V600E-Mutated Colorectal Cancer. *N Engl J Med.* 2019;381(17):1632-1643.
3. Overman MJ, Lonardi S, Wong KYM, et al. Durable Clinical Benefit With Nivolumab Plus Ipilimumab in DNA Mismatch Repair-Deficient/Microsatellite Instability-High Metastatic Colorectal Cancer. *J Clin Oncol.* 2018;36(8):773-779.

©2021 Patel KK et al. *JAMA Network Open*
